# A Crop Image Segmentation and Extraction Algorithm Based on Mask RCNN

**DOI:** 10.3390/e23091160

**Published:** 2021-09-03

**Authors:** Shijie Wang, Guiling Sun, Bowen Zheng, Yawen Du

**Affiliations:** College of Electronic Information and Optical Engineering, Nankai University, Tianjin 300350, China; sjwang@mail.nankai.edu.cn (S.W.); zhengbwen@mail.nankai.edu.cn (B.Z.); 2120190306@mail.nankai.edu.cn (Y.D.)

**Keywords:** Mask RCNN, instance segmentation, sobel operator, deep learning

## Abstract

The wide variety of crops in the image of agricultural products and the confusion with the surrounding environment information makes it difficult for traditional methods to extract crops accurately and efficiently. In this paper, an automatic extraction algorithm is proposed for crop images based on Mask RCNN. First, the Fruits 360 Dataset label is set with Labelme. Then, the Fruits 360 Dataset is preprocessed. Next, the data are divided into a training set and a test set. Additionally, an improved Mask RCNN network model structure is established using the PyTorch 1.8.1 deep learning framework, and path aggregation and features are added to the network design enhanced functions, optimized region extraction network, and feature pyramid network. The spatial information of the feature map is saved by the bilinear interpolation method in ROIAlign. Finally, the edge accuracy of the segmentation mask is further improved by adding a micro-fully connected layer to the mask branch of the ROI output, employing the Sobel operator to predict the target edge, and adding the edge loss to the loss function. Compared with FCN and Mask RCNN and other image extraction algorithms, the experimental results demonstrate that the improved Mask RCNN algorithm proposed in this paper is better in the precision, Recall, Average precision, Mean Average Precision, and F1 scores of crop image extraction results.

## 1. Introduction

In recent years, with the vigorous promotion of the concept of smart agriculture [1], traditional agricultural farming has been gradually abandoned, and agriculture has begun to develop in the direction of intelligence and automation. As early as the 1970s, image processing technology began to be applied to the agricultural field, mainly used in crop disease identification, phenotype detection, quality classification, etc. [2,3,4], which greatly improved agricultural farming efficiency and crop yield.

Image segmentation [5] is the process of decomposing the image into several regions according to the difference of the gray value of the image, and the researcher extracts the region of interest. As a key part of image processing, image segmentation has a great influence on the results of image analysis. Through image segmentation technology, crop information can be obtained efficiently and non-destructively, helping crop planters understand crop growth dynamics in real time, and better manage crops.

Weierstrass-Mandelbrot’s fractal function [6,7] can also overcome the instability of gray and edge features in complex natural scene images. Hyperspectral remote sensing technology is widely used in the fields of vegetation survey, remote sensing, agriculture, environmental monitoring, and atmospheric research [8,9]. There are huge opportunities and challenges regarding the analysis and processing of hyperspectral remote sensing images. How to retain more information on the basis of effectively removing redundancy in the process of image processing and analysis is the core of the current research [10,11].

Deep learning has made many breakthroughs and achievements in complex feature extraction and target recognition. Most current instance segmentation algorithms are based on candidate regions. Pinheiro et al. proposed the DeepMask segmentation model, which outputs a predictive candidate mask through the instances appearing in the input image to segment each instance object. However, its accuracy of boundary segmentation is low. Rendmon J et al. designed the YOLOV3 target Detection algorithm [12] to merge feature extraction and candidate frame prediction into a deep convolutional network through a newly designed residual network. Li et al. presented the first end-to-end instance segmentation framework, namely full convolution Instance Segmentation (FCIS) [13]. With the improvement of the position-sensitive score map, FCIS can predict a bounding box and instance segmentation, while it can only roughly detect the boundary of each instance object regarding overlapping object instances. Kaiming He et al. established the Mask RCNN target detection network model [14]. This model can fill pixels at non-integer positions in the feature map based on bilinear differences, so as to eliminate the position error when mapping high-dimensional feature maps to low-dimensional feature maps. As a result, the effect of target detection was significantly improved. This framework is an algorithm with relatively good instance segmentation results among existing segmentation algorithms.

If the category and location information of the object of interest in the image are determined on the example, the most popular target detection algorithms include RCNN, Fast RCNN, Faster RCNN, and U-net [15,16,17]. However, these frameworks require considerable training data and cannot achieve end-to-end detection. The positioning ability of the detection frame is limited. Moreover, gradients would disappear or explode often as the number of convolutional layers increases during the process of extracting features. In response to these shortcomings, He Kaiming et al. proposed a residual network (ResNet) that helps the model converge through the residual module and combined it with the target detection model Mask RCNN, contributing to accelerating the training speed of the neural network. The target detection and segmentation are realized, and the accuracy of model detection is remarkably improved. Mask RCNN is the first deep learning model connecting target detection and segmentation in one network. It can achieve challenging instance segmentation tasks, allowing it to not only accurately segment individuals in different categories but also mark each pixel in the image to distinguish different individuals in the same category.

The traditional Mask RCNN network is built based on the Keras deep learning framework with TensorFlow as the backend. Due to the constraints of the framework, the performance of the network cannot be brought into full play. Therefore, we adopted the Mask RCNN of the Pytorch framework, and optimized and tested the optimized Mask RCNN network on this basis. The performance improvement of the Mask RCNN network is under the new framework. The use of computer video memory resources is more efficient, and the speed and accuracy of calculations have also been significantly improved. Moreover, the new framework is not only easy to debug, it is highly modular, very convenient to build models, and the migration of data parameters between CPU and GPU is very flexible.

Therefore, this paper proposes a crop image extraction algorithm based on Mask RCNN, using the PyTorch deep learning framework, in which the Mask RCNN network model structure is improved, path aggregation and feature enhancement functions are added to the network design, the region extraction network (RPN) is improved, and the feature pyramid network (FPN) is optimized. Finally, a micro-fully connected layer is added to the mask branch of the ROI output, the Sobel operator is used to predict the target edge, and edge loss is added to the loss function, so as to further boost the edge accuracy of the segmentation mask. The experimental results in the Fruits 360 Dataset demonstrate that the improved Mask RCNN algorithm proposed in this paper has better performance in crop image extraction.

## 2. Mask RCNN Algorithm

Mask RCNN is an instance segmentation framework extended by Faster RCNN. It is divided into two stages: The first stage scans the image and generates suggestions, and the second stage classifies the suggestions and generates bounding boxes and masks.

### 2.1. Mask RCNN Network Structure

**Figure 1:**
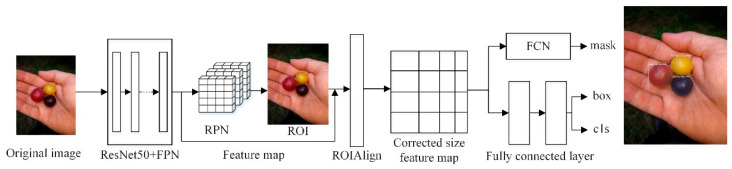
Mask RCNN algorithm network structure.

As an instance segmentation algorithm model, Mask RCNN can simultaneously perform pixel-level object segmentation and target recognition. The network structure of Mask RCNN not only inherits the original Faster RCNN network structure, but also introduces the feature pyramid network (FPN) and the region of interest alignment algorithm (ROIAlign). Its main structure is divided into six parts: Input, feature extraction backbone network, FPN, regional candidate network (RPN), ROIAlign and bounding box, category, and mask (Box, Class, Mask) output. The network structure diagram of Mask RCNN algorithm is shown in Figure 1.

In Mask RCNN, the image to be processed is first inputted into the pre-trained ResNet50 + FPN network model to extract features and obtain the corresponding feature maps. In turn, the feature map obtains a considerable number of candidate frames (regions of interest or ROI) through RPN. Then, binary classification of the foreground and background is performed using the SoftMax classifier [18]. More accurate candidate frame position information is obtained from frame regression. Additionally, part of the ROI is filtered out under non-maximum suppression. Afterwards, the feature map and the last remaining ROI are sent to the RoIAlign layer, enabling each ROI to generate a fixed-size feature map. Finally, the flow passes through two branches, one branch enters the fully connected layer for object classification and frame regression, and the other branch enters the full convolutional network (FCN) for pixel segmentation.

### 2.2. Mask RCNN Backbone Network and FPN

Generally, the backbone network of Mask RCNN uses ResNet101. Specifically, the number of network layers is 101. Too many layers will greatly reduce the speed of the network structure. Considering that the crop image extraction trained in this article is relatively simple, and has low requirements on the network layer, ResNet50 is employed in this paper to further improve the running speed of the algorithm.

Given the large difference in size caused by the different types of crops in the image, only one convolutional neural network cannot extract all the image attributes well. Therefore, the backbone structure of ResNet50 and the FPN feature pyramid network are adopted in this study. FPN uses a top-down hierarchical structure with horizontal connections, from single-scale input to building a network feature pyramid, solving the multi-scale problem of extracting target objects from images and requires fewer parameters.

**Figure 2:**
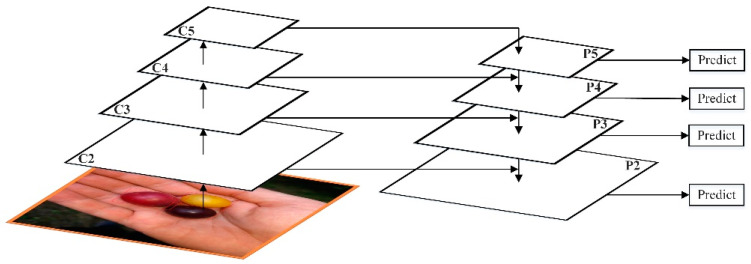
FPN network architecture.

FPN adopts the form of Feature Map in the pyramid of SSD. Unlike SSD, FPN not only uses the deep Feature Map in VGG [19], but the shallow Feature Map is also applied to FPN. Additionally, through bottom-up (bottom-up), top-down (top-down), and lateral connection (lateral connection), these Feature Maps are integrated efficiently, which improves the accuracy without significantly increasing the detection time. The FPN network architecture is shown in Figure 2.

Bottom-up is the forward process of the convolutional network. In the forward process, the size of the feature map will change after passing through some layers, but will not change when passing through some other layers. The feature map layer that has not changed is classified as a stage. Therefore, the features extracted each time are the output of the last layer of each stage, which can form a feature pyramid.

Through the bottom-up path, FPN obtains four sets of Feature Maps. The shallow Feature Map such as C2 contains more texture information, while the deep Feature Map such as C5 contains more semantic information. In order to combine these four groups of Feature Maps with different characteristics, FPN uses a top-down and horizontal connection strategy.

The C1–C5 obtained by the residual network have undergone different downsampling times, so the size of the feature map obtained is also different. In order to improve computational efficiency, FPN first uses 1 × 1 to reduce the dimensionality to obtain P5, and then uses bilinear interpolation for up-sampling, and up-samples P5 to the same size as C4.

After that, FPN also used 1×1 convolution to reduce the dimensionality of P4. Since dimensionality reduction does not change the size, P5 and P4 have the same size. FPN directly adds the unit of P5 to P4 to get the updated P4. Based on the same strategy, P4 is used to update P3, and P3 to update P2. This whole process is updated from the top layer of the network to the lower layer, which is called the top-down path.

FPN uses unit addition operations to update features. This unit addition operation is called horizontal connection. Since the unit addition is used, P2, P3, P4, and P5 should have the same number of Feature Maps. After fusion, a 3×3 convolution kernel will be used to convolve each fusion result in order to eliminate the aliasing effect of upsampling [20].

### 2.3. RoIAlign

In the Mask RCNN network structure, the mask branch must determine whether a given pixel is part of the target, and the accuracy must be at the pixel level. After a lot of convolutions and merging of the original image, the size of the image has been changed. The image target object cannot be accurately located when the pixel-level segmentation is performed directly. Thus, Mask RCNN is improved based on Faster RCNN, and the ROI Pooling layer is changed to the region of an interest alignment layer (RoIAlign). The bilinear interpolation method [21] preserves the spatial information on the feature map, avoiding the error caused by the two quantizations of the feature map in the ROI Pooling layer and handling the regional mismatch of the image object problem. Therefore, pixel-level detection segmentation can be achieved.

**Figure 3:**
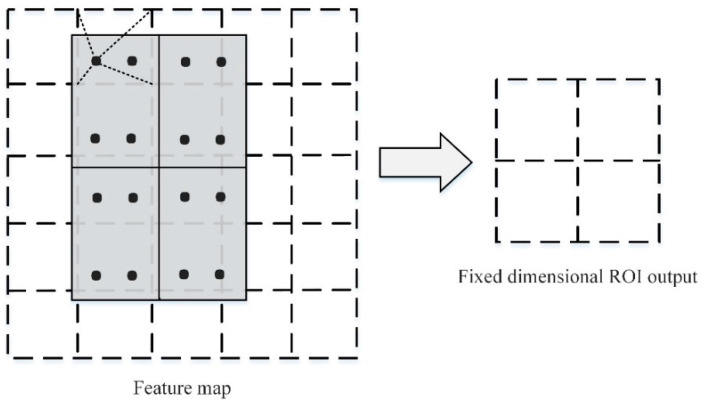
ROIAlign bipolar interpolation.

The difference between the ROI alignment layer RoIAlign and ROI merging is that it eliminates the quantization operation and does not quantize the ROI boundary and unit. Moreover, the exact position of the sampling point in each unit is calculated using bilinear interpolation while keeping the decimals. Afterwards, the maximum pooling or average pooling operation is employed to output the last fixed-size ROI. As illustrated in Figure 3, the black dashed line is the 5 × 5 feature map after convolution, and the solid line is the small feature block corresponding to the ROI in the feature map. RoIAlign maintains the floating-point number boundary without quantization processing. The small feature blocks are first divided into 2 × 2 units (each unit boundary is not quantized) and then divided into four small blocks in each unit. Next, the small feature blocks are divided into four small blocks. As exhibited by the blue dots in the figure, the center point is taken as the four coordinate positions. The values of the four positions are calculated by bilinear interpolation. Finally, the maximum merging or average merging operation is performed to obtain a 2 × 2 feature map.

## 3. Improved Mask RCNN Algorithm Based on Crop Image Extraction

In the crop image extraction, due to the large differences in the size of different crop types, it is difficult to extract all the features of the image by relying on a single convolutional neural network. Therefore, this paper adopts the backbone structure of ResNet50 and an FPN feature pyramid network, and solves the multi-scale problem of extracting target objects in the image through the backbone network and the FPN network connected horizontally from top to bottom.

The traditional Mask RCNN network is powerful, but the main module RPN of the network has the problem of a large amount of calculation and low efficiency. The path from low-level feature to high-level feature in the FPN network feature map is too long, which increases the positioning information. The difficulty of flow is not conducive to the effective integration of information. Moreover, the mask prediction is only performed on a single field of view, and more diversified information cannot be obtained. Additionally, the detection and segmentation effect for special targets is not good. In this paper, while inheriting the advantages of Mask RCNN, according to the particularity of agricultural products in remote crop images, the following improvements have been made to the network.

### 3.1. Improvement of RPN Network

In this article, the FPN structure is adopted to separate images into different sizes and generate features corresponding to different sizes. Shallow features and deep features can distinguish simple large targets and small targets, respectively. Feature maps of different sizes generated by FPN are inputted into RPN. Then, RPN can extract ROI features from different levels of the feature pyramid according to the size of the target object. In this way, the simple network structure is changed, the detection performance of small objects is significantly improved without greatly increasing the amount of calculation, and the accuracy and speed are extremely enhanced.

RPN is equivalent to a classless target detector based on a sliding window. It is based on the structure of a convolutional neural network. The sliding frame scans to generate frame anchors. It uses nine target frames with different areas and sizes to estimate the original target on each sliding window and the size of the location. The proposed area can generate a large number of anchor points of different sizes and aspect ratios, and they overlap to cover as many images as possible. The size of the proposed area and the amount of overlap (IOU) of the required area will directly affect the classification effect.

**Figure 4:**
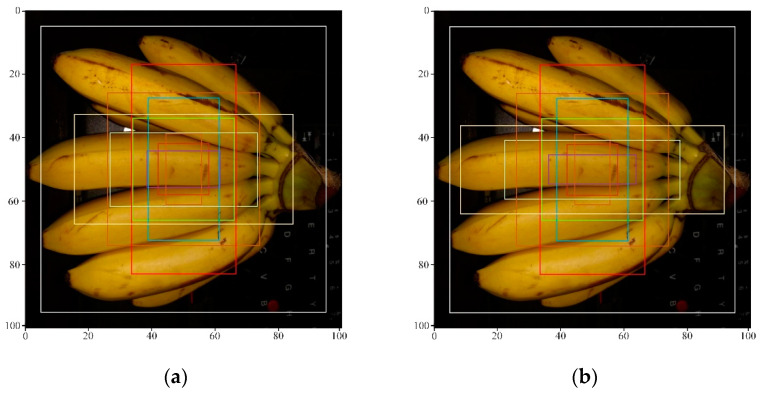
ROI generated before and after the RPN network improvement. (**a**) ROI generated before the RPN network optimization; (**b**) ROI generated after the RPN network optimization.

Without increasing the amount of calculation, the new aspect ratio anchor point conforms to the characteristics of the cropped graphics. Compared with the original Mask RCNN, the cropped image extraction is more accurate. In order to be able to adapt to more crop types, the algorithm adjusts the zoom ratio of the anchor point to {16×16, 32×32, 64×64, 256×256}, and the aspect ratio anchor point to {1:2, 1:1, 3:1}. The improved anchor frame ratio is more suitable for crop image extraction. The detection and segmentation effects of small targets can be improved, and the detection rate of target frames can be improved, as shown in Figure 4.

IOU is the coverage of the predicted box and the real box. Its value is equal to the intersection of the two boxes divided by the union of the two boxes. Without modifying this part of the network model, the IOU of the RPN network is almost the same as the empirical figure. In this article, the value of IOU is set to 0.6, indicating that it is the foreground when the overlap ratio between the area corresponding to the anchor frame and the actual target area is greater than 0.6, it is the background when the overlap ratio is less than 0.4. The number between the two values is discarded. This reduces the amount of calculation based on the model, saves calculation time, and improves the efficiency of the model.

### 3.2. Improvement of FPN Network and Mask Branch

The feature pyramid network is proposed to better realize the fusion of top-level and bottom-level feature maps and the effective use of features at various stages. Mask RCNN uses a top-down approach to mix high-level features with low-level features to improve all features in FPN that have reasonable classification capabilities.

FPN has proved that adding a top-down bypass connection can add high-level semantics to features to facilitate classification, but low-level features are more conducive to positioning. Although FPN middle-and high-level features such as P5 also have low-level features indirectly, the information flow route is too long and needs to go through the ResNet50 network.

**Figure 5:**
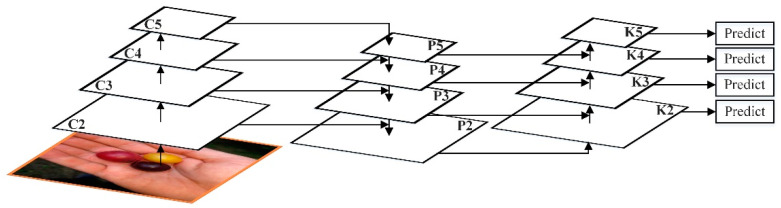
Improved FPN network structure.

This paper uses the bottom-up idea to further integrate features, so that the path for high-level features to obtain low-level features is shortened, and a clear lateral connection path is constructed from low-level to high-level, which is used to shorten the information path and make the bottom layer information flow faster. Moreover, a bottom-up enhancement path is created, and then the improved FPN network uses the precise positioning signal stored in the bottom-up low-level feature to enhance the feature pyramid architecture, as shown in Figure 5.

**Figure 6:**
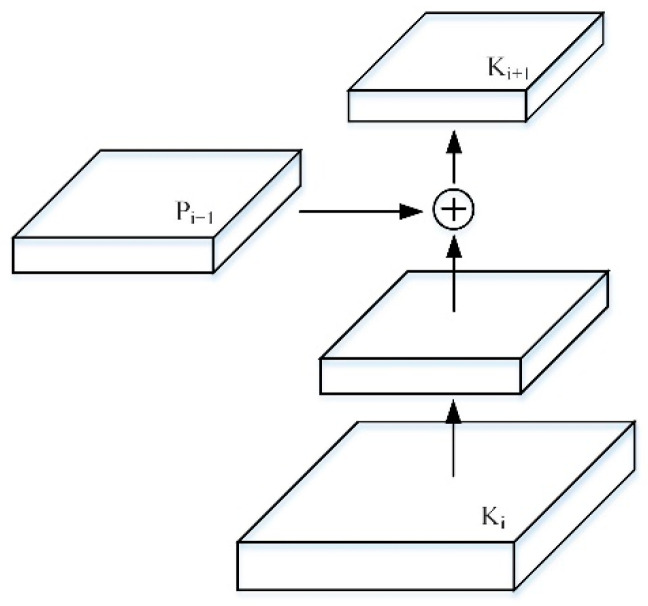
Bottom-up feature map structure.

Using $\left\{K_{2},K_{3},K_{4},K_{5}\right\}$ corresponds to the feature map generated by $\left\{P_{2},P_{3},P_{4},P_{5}\right\}$, where $K_{2}$ is directly copied by $P_{2}$ without any other processing. Each feature map connects the higher resolution $K_{i}$ and the lower resolution $P_{i+1}$ through the lateral connection to generate a new feature map $K_{i+1}$, as shown in Figure 6.

First, after each feature map in which $K_{i}$ undergoes a 3×3 convolution with a step size of 2, the resolution is reduced by two times, reducing the spatial size and making its own resolution consistent with $P_{i+1}$. Furthermore, each element of $K_{i}$ and $P_{i+1}$ after the down-sampling resolution is reduced, is added through the lateral connection. Finally, the resulting feature map undergoes a 3×3 convolution to obtain $K_{i+1}$. The bottom-up path aggregation of the FPN network is realized, and the positioning ability of the entire feature structure is further improved by propagating low-level responses.

In the crop extraction experiment, for small crops, the Mask RCNN network has better extraction results, and when extracting larger crops, the larger crops lose a lot of detailed information due to the high zoom ratio, and there are no mask boundaries, which are too complete and precise.

The component responsible for predicting the mask in the Mask RCNN network is a lightweight and easy-to-implement branch. The input of the mask branch is the pooled feature grid after each candidate region is fused. The traditional main branch is four consecutive convolutional layers and a deconvolution layer. Among them, the kernel size of each convolutional layer is 3×3 with 256 channels, followed by a deconvolutional layer with up-sampling of 2 times to predict each category.

**Figure 7:**
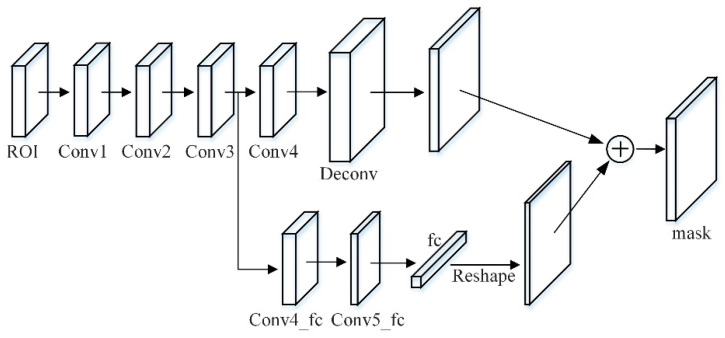
Adding of micro fully connected layer branch to the mask.

In this paper, a branch of the micro fully connected layer is added to the mask branch, which is connected from conv3 to the fully connected layer through a branch, passing two conv4_fc and conv5_fc with a 3×3 size of 256 channels. Among them, the number of conv5_fc convolutional layer channels is halved to reduce the amount of calculation, and the mask size is set to 28×28. The 784×1×1 vector generated by the fully connected layer is reshaped to the same spatial size as the mask predicted by FCN, and finally added to the output of FCN to obtain the final prediction. The fully connected layer is used to predict unknowable background or foreground masks. It is not only efficient, but also allows more samples to train the parameters of the fully connected layer. Additionally, it has stronger generalization ability and only one fully connected layer is used, which avoids hiding spatial features. A problem of collapsing into short eigenvectors is shown in Figure 7.

In order to capture the different views proposed by each feature, a tiny fully connected layer is used to increase the mask prediction. The fully connected layer and the original FCN have complementary characteristics. By fusing the predictions of these two views, the diversity of information will increase to achieve feature enhancement and produce better quality mask effects.

### 3.3. Optimization Based on the Segmentation Loss Function

The loss function of Mask RCNN consists of three parts: Classification error, regression error, and segmentation error as shown in Equation (1):

(1) $$L=L_{cls}+L_{box}+L_{mask}$$

The above formula is the same as the loss function in the Faster RCNN model, representing the classification error and the detection error, respectively. Decoupling the mask branch and the class prediction branch is the average binary cross-entropy loss. A binary mask is independently predicted for each category, and does not depend on the prediction result of the classification branch. The loss function in Faster RCNN is expressed as Equation (2):

(2) $$L(\{p_{i}\},\{t_{i}\})=\frac{1}{N_{cls}}\sum_{i}L_{cls}(p_{i},p_{i}^{*})+\lambda \frac{1}{N_{reg}}\sum_{i}p_{i}^{*}L_{reg}(t_{i},t_{i}^{*})$$

In the above formula, i is the index of the anchor box in the mini-batch, $N_{cls}$ and $N_{reg}$ represent the number of classification layers and the number of regression layers, respectively, $P_{i}$ represents anchor i, which is the predicted probability value of the object. If the anchor box is negative, then $P_{i}^{*}$ is 0, if the anchor box is positive, then it is 1, $t_{i}$ represents the four parameterized coordinates of the prediction candidate box, $t_{i}^{*}$ refers to the four parameterized coordinates of the true value region, $L_{cls}$ and $L_{reg}$ represent the classification loss and regression loss, respectively. In addition, λ stands for the balance coefficient, which is used to control the ratio of the two loss functions.

In Faster RCNN, due to the large gap between $N_{cls}$ and $N_{reg}$, a hyperparameter λ control balance is introduced between the classification loss and the regression loss. In general $N_{cls}=256$, $N_{reg}=2400$, so λ=10 is usually set, and large-scale targets and small-scale targets share this parameter. The hyperparameter λ=10 is still introduced in Mask RCNN.

In order to further improve the accuracy of the segmentation mask, a method of adding edge loss [22] to the mask branch is proposed to make the edge of the segmentation result more accurate. First, the labeled image is converted into a binary segmentation map of the crop, which is the target mask, and then the prediction mask and the target mask output by the mask branch are used as input, and they are convolved with the Sobel operator [23]. The Sobel operator is a two-dimensional operator such as Equations (3) and (4). The mean square error of the convolution result is calculated, and the edge loss $L_{edge}$ is obtained as Equation (5):

(3) $$S_{x}=\left[\begin{array}{ccc} 1 & 0 & -1\\ 2 & 0 & -2\\ 1 & 0 & -1 \end{array}\right]$$

(4) $$S_{y}=\left[\begin{array}{ccc} 1 & 2 & 1\\ 0 & 0 & 0\\ -1 & -2 & -1 \end{array}\right]$$

(5) $$L_{edge}=\frac{1}{2}(y-{\hat{y}}^{2})$$

where y represents the labeled target edge, $\hat{y}$ represents the predicted edge, and the final improved loss function is shown in Equation (6):

(6) $$L_{total}=L_{cls}+L_{box}+L_{mask}+L_{edge}$$

The Sobel operator separately describes the horizontal and vertical gradients. During the gradient descent process during training, not only the edge strength along the x and y axes can be used, but also the direction of the edge can be used to minimize the total loss. This additional information can speed up training and reduce a lot of training time.

## 4. Experimental Results and Analysis

This experiment uses the open source PyTorch learning framework, Python language programming to realize the algorithm network. In addition, the hardware environment is Dell T5820 workstation from Tianjin, China, equipped with dual NVIDIA Quadro P4000 graphics cards (8 GB), 64-bit Ubuntu16.04 operating system.

PyTorch is an open source neural network framework first launched by Facebook in early 2017. Mask RCNN Benchmark is a fast and modular Faster RCNN and Mask RCNN component written entirely by PyTorch. This component is designed to make it easier for users to create a model to realize the recognition and segmentation of the target in the picture. The process is as follows: Use the Mask RCNN Benchmark open source project, combined with the pre-processed Fruits 360 Dataset, through supervised learning and migration learning [24], combined with the network’s pre-training weights to train the network, save the final training weights, and use the prediction function to predict and segment. In this way, the crops in the images of the Dataset can be accurately extracted.

### 4.1. Dataset Production

Images showing crops are detected and processed using transfer learning and Mask RCNN to reduce the number of steps in making Dataset labels and improve the detection accuracy of crop images.

Although deep learning requires a lot of data, it is difficult to find enough training data for a specific problem within a certain range in most cases. A solution, namely transfer learning, is proposed to solve this problem.

Transfer learning includes the source domain and target domain, defined as Equation (7):

(7) D(s)={x,P(x)}, D(t)={x,P(x)}

where D(s) denotes the source domain, D(t) indicates the target domain, x represents the feature space, P(X) refers to the marginal probability distribution, and X satisfies Equation (8):

(8) $$X=\{x_{1},K,x_{n}\}\in x$$

It can be seen that migration learning is used to migrate model parameters that have been trained in the source domain to a new model in the target domain in order to help the training of the new model. This article first conducts pre-training on a large coco Dataset, and then transfers the trained weight files to the dedicated Dataset collected in this article for training and fine-tuning the network parameters. This enables convolutional neural networks to achieve good results on small Datasets, thereby alleviating the problem of insufficient data sources.

**Figure 8:**
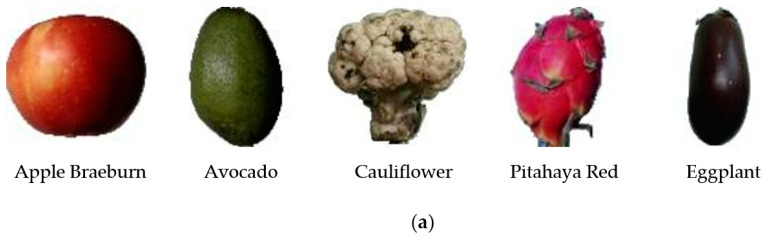

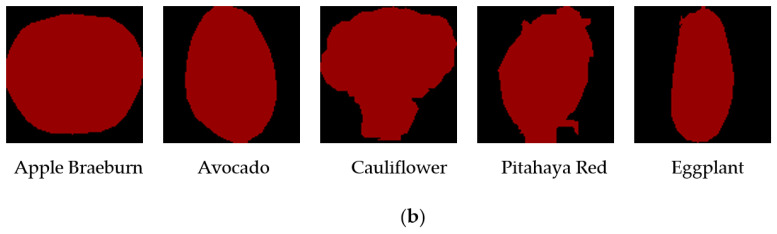
Image samples of some crops in the Labelme annotated Dataset: (**a**) Original image; (**b**) manual segmentation mask map.

The crop image data source used in this experiment is the Kaggle open source Dataset Fruits 360, which contains 131 kinds of fruits and vegetables, a total of 90,483 images, and the image size is 100 × 100 pixels. Among them, there are a total of 103 images of various types of crops. The Fruits 360 Dataset test set contains 67,692 images of a single crop type, and the test set contains 22,688 images of a single crop type. Due to the large number of images in the Dataset, image enhancement and other processing are not required. The image Datasets in this article are all labeled by Labelme, and the generated json files are converted to coco format and trained on the Pytorch platform. Some image samples after Labelme labeling are shown in Figure 8.

The labeling tool Labelme is used to label the crop images of the Fruits 360 Dataset. Due to the huge number of images in the Dataset, manual labeling takes a long time, and the images in the Dataset are all single crop types. The crop image gaps of the same category are small, and data training is required, which is a json file rather than a point image.

This article uses Labelme to label several pieces of each category in the Dataset, uses Python scripts for feature area detection and corresponding APIs, modifies the value of the key corresponding to the json dictionary index, and uses the movement distance of the feature points to adjust the shape of the label box and the relative image distance. To generate json files, Labelme’s independent labeling of Datasets are realized, which greatly reduces the time wasted in Dataset production.

### 4.2. Evaluation Index

In order to quantitatively evaluate the comprehensive performance of the crop image extraction algorithm based on Mask RCNN, this paper adopts Recall, Precision, Average precision (AP), Mean average precision (mAP), and F-measure ($F_{1}$) [25,26] as the evaluation of crop image extraction index. Each category in the Dataset can draw a curve based on precision and recall. Average precision (AP) is the area enclosed by the curve and the coordinate axis. In addition, mAP is obtained by averaging the AP values of all categories. The indicators are similar to Equations (9)–(13):

(9) $$P=\frac{T_{p}}{T_{p}+F_{p}}\times 100\%$$

(10) $$R=\frac{T_{P}}{T_{P}+F_{N}}\times 100\%$$

(11) $$AP=\int_{0}^{1}P(R)dR$$

(12) $$mAP=\frac{\sum AP}{n}$$

(13) $$F_{1}=\frac{2\times P\times R}{P+R}$$

where $T_{P}$ is the number of positive samples that are correctly predicted, $F_{P}$ is the number of samples where negative samples are predicted to be positive samples, $F_{N}$ is the number of samples where positive samples are predicted to be negative samples, and n is the number of Dataset categories.

In order to verify that the method in this paper has greater advantages with mainstream extraction algorithms, the Fruits 360 Dataset is tested with algorithms based on FCN, U-net, Mask RCNN, etc.

### 4.3. Data Verification

In order to test the stability of the improved Mask RCNN model more accurately, we first extracted 1000 Fruits 360 Dataset images for training, of which 800 images are the training set, 100 images are the validation set, and 100 images are the test set. At the same time, there are 20 multi crop images in the verification set and the test set, respectively.

We performed an analysis to evaluate the robustness of the improved Mask RCNN framework in crop image detection by comparing the improved Mask RCNN framework with other methods. To achieve this, the accuracy of the improved Mask RCNN was compared with other basic models, namely FCN, U-net, and Mask RCNN.

Table 1 shows the comparative analysis of the proposed method and other frameworks in terms of model parameters and detection accuracy. The results of this comparative analysis show that the improved Mask RCNN performs better than FCN, U-net, and Mask RCNN.

In addition, as can be seen from Table 1, FCN has the highest model parameters and is the most expensive method to execute. On the contrary, the framework of the improved Mask RCNN model is the most economically efficient, with an execution time of only 1482 s. The main reason for the efficient performance of the improved Mask RCNN is that the anchor frame selection is faster, and the higher-level response of FPN can more accurately locate low-level information. This architecture uses the effective reuse of edge loss and improves the extraction of image edge information.

**Figure 9:**
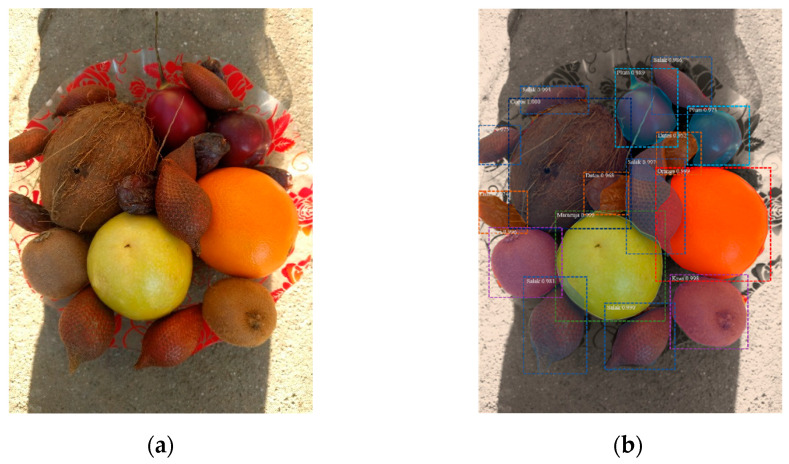

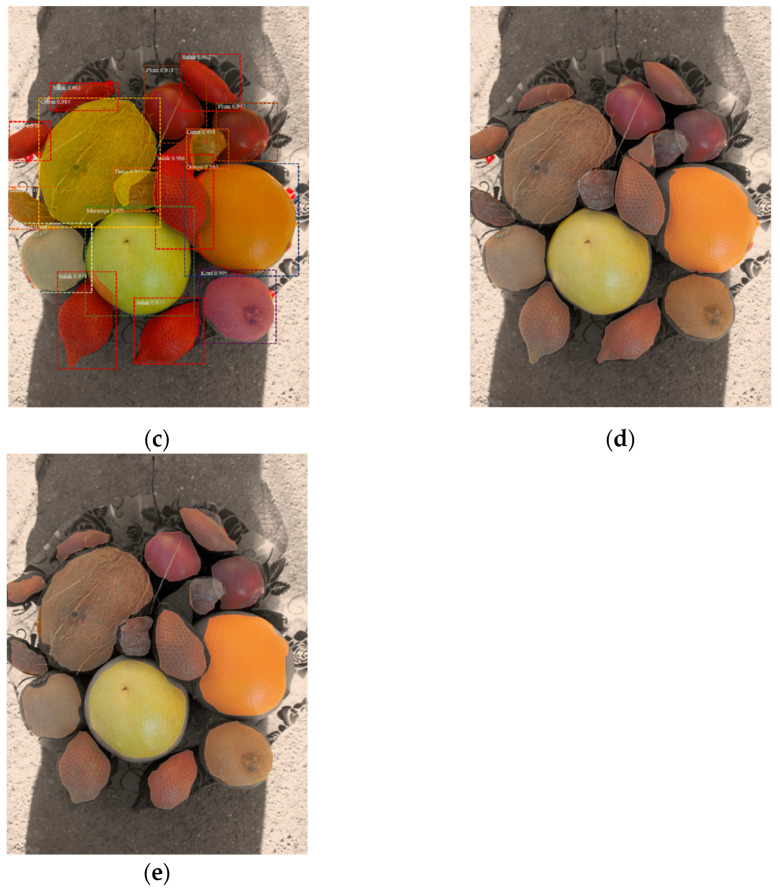
Image segmentation of multiple crops: (**a**) Original image; (**b**) method of this article; (**c**) Mask RCNN; (**d**) U-net; (**e**) FCN.4.4.

It can be concluded from Figure 9 that the economic cost of the comparison technique is high, and it cannot show an effective classification performance for samples with noise, blur, scale, and angle changes. From the analysis performed, it can be concluded that the improved Mask-RCNN we used has better performance than other deep learning models in terms of accuracy and effectiveness. The segmentation effect of multiple types of crops is shown in Figure 9.

#### Analysis of Results

**Figure 10:**
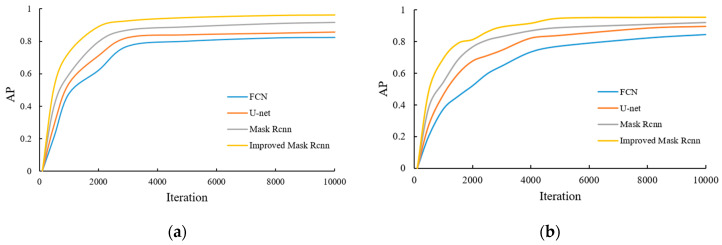

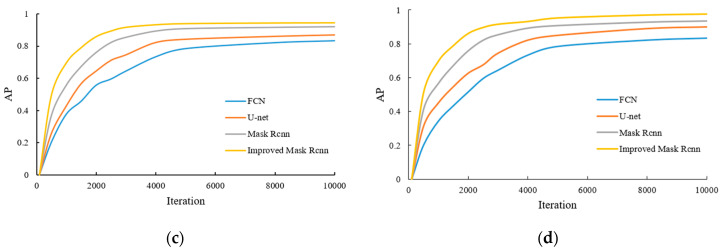
AP value of some crops: (**a**) Beetroot; (**b**) Granadilla; (**c**) Kaki; (**d**) Onion White.

During the model training process, the AP value of each category is recorded and mAP is calculated. The results of some categories of AP are shown in Figure 10.

The AP and mAP values of some crops obtained by the algorithm of this paper and FCN, U-net, and Mask RCNN algorithms are shown in Table 2.

It can be concluded from Figure 10 and Table 2 that as the model training continues to deepen, the AP value of each category gradually increases and reaches a higher stable value. Compared with the FCN, U-net, and Mask RCNN algorithms, the mAP value of the improved Mask RCNN reaches 0.949, which has a higher average accuracy of detection results, and can achieve target segmentation and extraction more quickly in the iterative process. This indicates that the performance of the proposed method is excellent and the effectiveness of target recognition and segmentation is significantly improved.

Table 3 shows the comprehensive indicators of the algorithm in this paper and the FCN, U-net, and Mask RCNN image extraction algorithms in the Fruits 360 Dataset. The resolution of the Fruits 360 Dataset is low, which tests the feature extraction performance of the algorithm in terms of image extraction. The improved Mask RCNN algorithm proposed in this paper has a mAP value of 0.895 and an F-measure value of 0.913, which is 8.8% and 7.9% higher than the Mask RCNN algorithm.

**Figure 11:**
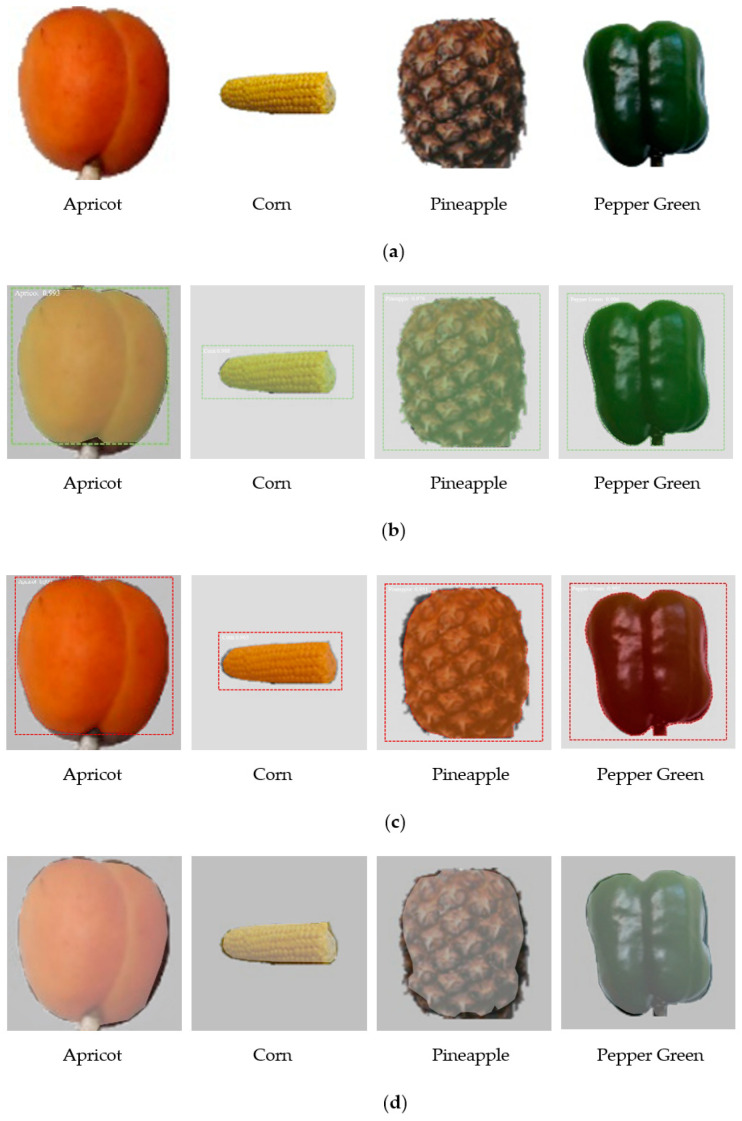

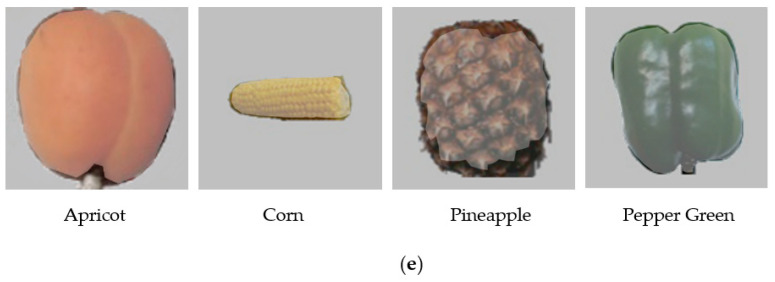
Example of partial crops segmentation: (**a**) Original image; (**b**) method of this article; (**c**) Mask RCNN; (**d**) U-net; (**e**) FCN.

The segmentation results of some types of crops are shown in Figure 11. It can be seen from Figure 11 that at lower resolution, the FCN algorithm does not perform well in crop segmentation. When segmenting and extracting pineapple images, both U-net and Mask RCNN algorithms have unsatisfactory segmentation results. However, the algorithm in this paper still maintains a good segmentation effect. In a comprehensive comparison, the high scores of the two evaluation indicators of mAP and F1 indicate that the performance of the method in this paper is excellent, and the accuracy, robustness, and generalization performance of the network have better performance.

## 5. Conclusions

In this paper, an automatic crop image extraction algorithm based on Mask RCNN is proposed to improve the accuracy of crop image segmentation and extraction. Using the PyTorch deep learning framework, the Mask RCNN network model structure is improved, the path aggregation and feature enhancement functions are added to the network design, the region extraction network (RPN) is improved, and the feature pyramid network (FPN) is optimized. In addition, the spatial information of the feature map is saved by the bilinear interpolation method in ROIAlign. Finally, a micro-fully connected layer is added to the mask branch of the ROI output, the Sobel operator is used to predict the target edge, and edge loss is added to the loss function, so as to further boost the edge accuracy of the segmentation mask.

Compared with image extraction algorithms such as FCN, U-net, and Mask RCNN, the experimental results demonstrate that the improved Mask RCNN algorithm proposed in this paper has better performance in crop image extraction. Since the image pixels of the Dataset selected in this article are small, there is room for further improvement in anchor construction. Additionally, the shallow semantic information of the feature network will be further integrated in more in-depth research.

## Figures and Tables

**Table 1:** Comparative analysis of the proposed approach with other models.

Parameters	FCN	U-net	Mask RCNN	Improved Mask RCNN
Training accuracy	89.71%	94.23%	98.86%	99.83%
Val accuracy	88.95%	92.11%	97.99%	99.68%
Test accuracy	86.49%	91.52%	97.38%	99.66%
Processing time (s)	4357	2504	1326	1482

**Table 2:** AP and mAP values of some crops.

Module	mAP	AP			
		Beetroot	Granadilla	Kaki	Onion White
FCN	0.834	0.823	0.844	0.836	0.834
U-net	0.882	0.857	0.898	0.870	0.901
Mask RCNN	0.929	0.936	0.922	0.919	0.937
Improved Mask RCNN	0.949	0.948	0.952	0.944	0.951

**Table 3:** Overall Dataset algorithm comparison.

Module	FCN	U-net	Mask RCNN	Improved Mask RCNN
mAP	0.753	0.792	0.822	0.895
$F_{1}$	0.761	0.814	0.846	0.913

## Data Availability

Publicly available datasets were analyzed in this study. This data can be found here: https://www.kaggle.com/moltean/fruits (access on 1 August 2021).

## References

[1] Uddin M.A., Ayaz M., Mansour A., Aggoune E.M., Sharif Z., Razzak I. (2021). Cloud-connected flying edge computing for smart agriculture. Peer Peer Netw. Appl..

[2] Agarwal M., Gupta S.K., Biswas K.K. (2020). Development of Efficient CNN model for Tomato crop disease identification. Sustain. Comput. Inform. Syst..

[3] Iraji M.S. (2019). Comparison between soft computing methods for tomato quality grading using machine vision. J. Food Meas. Charact..

[4] Pratapa A., Doron M., Caicedo J.C. (2021). Image-based cell phenotyping with deep learning. Curr. Opin. Chem. Biol..

[5] Minaee S., Boykov Y.Y., Porikli F., Plaza A.J., Kehtarnavaz N., Terzopoulos D. (2021). Image segmentation using deep learning: A survey. IEEE Trans. Pattern Anal. Mach. Intell..

[6] Guariglia E. (2016). Entropy and fractal antennas. Entropy.

[7] Berry M.V., Lewis Z.V., Nye J.F. (1980). On the weierstrass-mandelbrot fractal function. Proc. R. Soc. Lond. A Math. Phys. Sci..

[8] Yang L., Su H.L., Zhong C., Meng Z.Q., Luo H.W., Li X.C., Tang Y.Y., Lu Y. (2019). Hyperspectral image classification using wavelet transform-based smooth ordering. Int. J. Wavelets Multi..

[9] Guariglia E. (2018). Harmonic sierpinski gasket and applications. Entropy.

[10] Guariglia E., Silvestrov S. (2016). Fractional-wavelet analysis of positive definite distributions and wavelets on $$\varvec {\mathscr {D’}}(\mathbb {C}) $$. Engineering Mathematics II..

[11] Zheng X.W., Tang Y.Y., Zhou J.T. (2019). A framework of adaptive multiscale wavelet decomposition for signals on undirected graphs. IEEE Trans. Signal Proces..

[12] Redmon J., Farhadi A. (2018). Yolov3: An incremental improvement. arXiv.

[13] Li Y., Qi H., Dai J., Ji X., Wei Y. Fully convolutional instance-aware semantic segmentation. Proceedings of the IEEE Conference on Computer Vision and Pattern Recognition.

[14] He K., Gkioxari G., Dollár P., Girshick R. Mask r-cnn. Proceedings of the IEEE International Conference on Computer Vision.

[15] Su W.H., Zhang J.J., Yang C., Page R., Szinyei T., Hirsch C.D., Steffenson B.J. (2021). Automatic evaluation of wheat resistance to fusarium head blight using dual mask-rcnn deep learning frameworks in computer vision. Remote. Sens.

[16] Ren Y., Zhu C.R., Xiao S.P. (2018). Object detection based on fast/faster rcnn employing fully convolutional architectures. Math. Probl. Eng..

[17] Aghabiglou A., Eksioglu E.M. (2021). Projection-based cascaded U-net model for MR image reconstruction. Comput. Methods Programs Biomed..

[18] Wu Q.H. (2020). Image retrieval method based on deep learning semantic feature extraction and regularization softmax. Multimed. Tools Appl.

[19] Abiram R.N., Vincent P.M.D.R. (2021). Identity preserving multi-pose facial expression recognition using fine tuned VGG on the latent space vector of generative adversarial network. Math. Biosci. Eng..

[20] Kipping D. (2021). The exomoon corridor: Half of all exomoons exhibit TTV frequencies within a narrow window due to aliasing. Mon. Not. R. Astron. Soc..

[21] Gui J.W., Wu Q.Q. (2020). Vehicle movement analyses considering altitude based on modified digital elevation model and spherical bilinear interpolation model: Evidence from GPS-equipped taxi data in Sanya, Zhengzhou, and Liaoyang. J. Adv. Transp..

[22] Zimmermann R.S., Siems J.N. (2019). Faster training of Mask R-CNN by focusing on instance boundaries. Comput. Vis Image Und.

[23] Tian R., Sun G.L., Liu X.C., Zheng B.W. (2021). Sobel edge detection based on weighted nuclear norm minimization image denoising. Electronics.

[24] Chouhan V., Singh S.K., Khamparia A., Gupta D., Tiwari P., Moreira C., Damasevicius R., de Albuquerque V.H.C. (2020). A novel transfer learning based approach for pneumonia detection in chest X-ray images. Appl. Sci..

[25] Revaud J., Almazán J., Rezende R.S., Souza C.R.d. Learning with average precision: Training image retrieval with a listwise loss. Proceedings of the IEEE/CVF International Conference on Computer Vision.

[26] Powers D.M. (2020). Evaluation: From precision, recall and F-measure to ROC, informedness, markedness and correlation. arXiv.

